# Association of aneurysmatic subarachnoid hemorrhage rate with environmental changes or emotional bursts

**DOI:** 10.1186/s41016-023-00322-7

**Published:** 2023-04-01

**Authors:** Athanasios K. Petridis, Igor Fischer, Humajoun Maslehaty

**Affiliations:** 1 Heinrich Heine University, Medical School, Dusseldorf, Germany; 2 Department of Neurosurgery, St. Luke Hospital, Thessaloniki, Greece; 3 Informatics and Data Science, Department of Neurosurgery, Heinrich Heine University, Moorenstr. 5, 40225 Dusseldorf, Germany; 4 Department of Neurosurgery, St. Vinzenz-Hospital, Dinslaken, Germany

**Keywords:** Brain aneurysm, Subarachnoid haemorrhage, Emotional burst

## Abstract

**Introduction:**

In the present letter we share the results of an analysis of more than 140,000 non traumatic arterial subarachnoid hemorrhages whereas the majority of them is expected to be after aneurysm rupture, in which we investigate a possible correlation of climatic changes and emotional bursts as correlating factors for such a rupture.

**Methods:**

We obtained the daily number of SAH from 2006 to 2018 for males and females from the German National statistics agency. The ICD codes provided to us were I60.1-I60.7, which are SAHs originating from intracranial arteries and excluding traumatic SAH and other not specified SAH.

**Results:**

An increase of mean SAH per day could be seen in winter compared to summer and family events seemed to have a protective effect against aneurysmal SAH. Additionally 6.55 more women per day suffer an SAH compared to men.

**Conclusion:**

There is a statistical significant higher risk of aneurysm ruptures in winter and in females, and a statistical lower number in Mother’s day.

**Supplementary Information:**

The online version contains supplementary material available at 10.1186/s41016-023-00322-7.

Dear Editor,

The question of aneurysmatic subarachnoid hemorrhage incidents and dependence of weather conditions or even other emotional stimulating events, has been risen during our daily hospital routine. In order to provide an answer to this questions, which is independent of the observations of one center or even one small geographical area, we present the results of our German wide analysis. The German national statistical agency (Statistisches Bundesamt, Destatis, Wiesbaden, 2019) provided us with the daily numbers of SAH ( primary diagnosis, non traumatic SAHI60* (I60.1-I60.7, SAH originating from middle-, anterior communicating-, posterior communicating-, basilar-, vertebral, other intracranial- and not further specified intracranial-arteries respectively)), from January 1st 2006 until December 31st 2018. We excluded I60.8, which are other non traumatic SAH and I60.9, which are unspecified non traumatic SAH. These data include all arterial/aneurysmatic non traumatic SAH (referred here as SAH), which means that there is only a minority of SAH classified as arterial although non aneurysmatic. These minority could not be separated from the aneurysmal SAH, but even with its inclusion we did not expect statistical deviation, which would change the conclusions of the aneurysmatic SAH, which is the great majority of the database. Still this could be a confounder and belongs to the limitations of the study [1]. Since the changes in SAH rates are not higher than on days with no holidays or other events, we can claim that there is no differences in SAH bleeding between our parameters. We assume that non aneurysmatic and aneurysmatic SAH changes are equal, especially under the expectant circumstances that the non aneurysmatic SAH, which received one of the above I60* ICD-10 codes are very few.

Statistical analysis was performed with Welch two sample t-test and the R-programe.

In summary 142,486 SAH cases were recorded through these years. The numbers for females and males were included. The mean bleeding rate for both genders together was 30 (standard deviation: 8), per day. The mean bleeding rate for every single day for females was 18.3 SAHs (SD of 6.1) and for males 11.7 (SD of 4.4). As shown in Fig. 1A, 6.55 females more than males suffer an SAH (*p* < 0.001). Another interesting observation apart from the question we seek to answer, is that a decrease of 0.6% of SAH for both genders can be seen for every single year from 2006 until 2018 (Fig. 1B). Table 1 shows the statistical analysis of all special events, which can be seen summarised in a graph in Fig. 1C (athletic events) and 1d (holidays). There is a protective effect on SAH in mother´s day (*p* < 0.01), and New Year’s Eve, Christmas, (less SAHs) and Olympic Summer games with a *p* < 0.001. Carnival seems to be associated with an increase of SAH events (*p* < 0.001). In a soccer nation like Germany, there was no difference of the mean number of SAHs during the World and European Soccer Championship. Not even when the German team was playing.

**Fig. 1:**
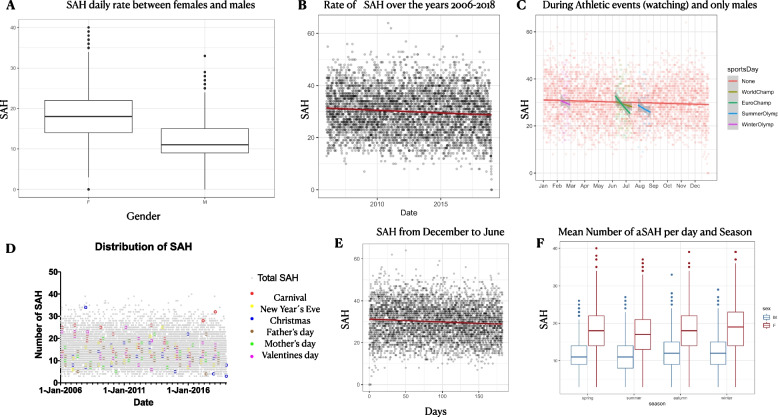
Aneurysmatic / arterial SAH rate and association to external and internal events. German nationwide Daily cases of SAHs are selected for males and females from 2006 to 2018. **A** Average of SAH cases per day in females (F) and males (M). There are 6.55 more females every day who suffer from SAH compared to males (*p* < 0.001). **B** Over the years of 2006 until 2018 there is a stable decline of SAH rate of 0.6% per year. **C** Graph shows that in athletic events there are not significant difference in aSAH numbers compared to other days, with exception of the Summer Olympics, which seem to have a decreased rate of SAH. **D** The number of SAH is not significantly different in different family events or holidays in first sight. The statistical analysis though shows a protective effect of mothers day and an increase for SAH in Carnival, i.e., **E** There is a decline of the daily average of SAH cases from December (0 days) to June (> 150 days) for every year (*p* < 0.001). **F** There is a significantly higher number of SAH in winter compared to summer in both genders

**Table 1:** Statistical analysis of the factors which could influence the rate of aSAH

Coefficients						
	Estimate	Std. error	z	value	Pr( >|z|)	
(Intercept)	3,446E + 03	5,459E + 00	63	1.278	< 2e − 16	***
New Year’s day	-4,656E − 01	5,435E − 02	–	8.568	< 2e − 16	***
Carnival	2,012E + 02	4,563E + 01		4.409	1.04e − 05	***
New Year´s Eve	-3,172E + 02	5,828E + 01	–	5.443	5.25e − 08	***
Christmas	-2,458E + 02	5,631E + 01	–	4.366	1.27e − 05	***
Valentine’s day	8,408E + 00	5,058E + 01		166	0.86798	
Father’s day	-2,236E + 02	5,73E + 01	–	3.903	9.50e − 05	***
Mother’s day	-1,599E + 02	5,542E + 01	–	2.884	0.00392	**
World soccer Championship	1,03E + 01	1,736E + 01		593	0.55314	
European soccer Championship	1,444E + 01	2,157E + 01		670	0.50317	
Olympic summergames	-7,737E + 01	2,715E + 01	–	2.849	0.00438	**
Olympic wintergames	-3,131E + 01	2,265E + 01	–	1.382	0.16682	

Signif. codes: 0 ‘***’ 0.001 ‘**’ 0.01 ‘*’ 0.05 ‘.’ 0.1 ‘ ‘ 1

A second question we would like to answer, was a possible seasonality associated with increased SAH rate. In fact there is a statistical significant decrease of SAHs from December to June, or from winter to summer (*p* < 0.001) (Fig. 1e, f). In males, the SAH average number per day was 12.08 compared to summer, where it was 11.39 (*p* < 0.001). In females, the average SAH rate per day in winter was 18.87, in spring 18.29 compared to summer where it was 17.69 (*p* < 0.001 for spring and winter compared to summer). In order to specify possible factors, which explain further this seasonality, we analysed the role of temperature, air pressure and humidity in association with SAH rates. As is shown in Supplemental Figure S1a, temperature is a significant factor for SAH in both genders. There is a significant decrease of the number of daily SAH when the temperature is rising from − 10° to + 30° Celsius (*p* < 0.001). Humidity too, plays a role in SAH rates. Supplemental Fig. 1b shows that dry climate is associated with higher number of SAH cases per day (males, *p* < 0.1 and females *p* < 0.001).

With this big data analysis we can safely reach conclusions about seasonality and emotional events, which can be associated with aneurysmatic SAH and incidence. The differences in the numbers of events are statistically significant, since the number of cases for 4745 days (13 years) allows to identify differences, which can be small but still statistical significant. A recent study indicates hypertension, as well as arguing or quarrelling and seasonality (autumn) as a risk factors of aneurysm rupture [2]. Carnival is such a period of arguing and quarreling, where we see a rise of SAH. It seems that in significant sports events the triggers for SAH is not high enough to induce such bleeding.

As can be seen in our study, the difference in SAH is about 1 patient less per day in Summer, or Mother’s day, which will not be realised in the clinical daily routine. But still the feeling clinicians have that more SAH/aSAH occur in winter in example, is true. We also have an indication that holidays, which are family oriented, like Christmas, are associated with less SAH cases, whereas holidays associated with high alcohol consumption in a cold weather can be associated with higher rates of SAH. Sports events do not seem to play a crucial role in SAH rates. The emotional burst of a lost or won soccer game, is not high enough to induce an SAH. It is also interesting that the yearly rate of the analysed types of aSAH from 2006 to 2018 is decreasing by 0.6% per year, which has also been observed in a Canadian study of 19765 patients, where a yearly decrease of 0.67% per year (from 2004 to 2015) could be seen [3]. This could be explained be earlier diagnosis and treatment of brain aneurysms or with a change in lifestyle, like less smoking, more athletic activities, earlier hypertensive treatment.

With this big data analysis of more than 140.000 cases we prove that aneurysm ruptures (and other non traumatic arterial SAHs) are dependent of some environmental and emotional changes, but that the difference, although statistically significant, would not be enough to be felt in clinical daily routine setting.

## Supplementary Information

**Additional file 1: Supplemental Figure S1.** Environmental factors associated with seasons of the year and SAH rate. **A **A rise of external (environmental) temperature is associated with a decrease of SAH rate. **B **Higher humidity leads to decrease of SAH rate. **C **Air pressure differences are not influencing the rate of SAH.

## Data Availability

The datasets used and/or analyzed during the current study are available from the corresponding author on reasonable request.

## Abbreviations

aSAH: Aneurysmatic subarachnoid hemorrhage
SAH: Nontraumatic aneurysmatic / arterial subarachnoid hemorrhage
