# Supplementary figures and images for: Association of aneurysmatic subarachnoid hemorrhage rate with environmental changes or emotional bursts
Source: Chin Neurosurg J. 2023 Apr 1;9:8. doi: 10.1186/s41016-023-00322-7 (PMC10067275; doi:10.1186/s41016-023-00322-7)

**A** Temperature and aSAH rate

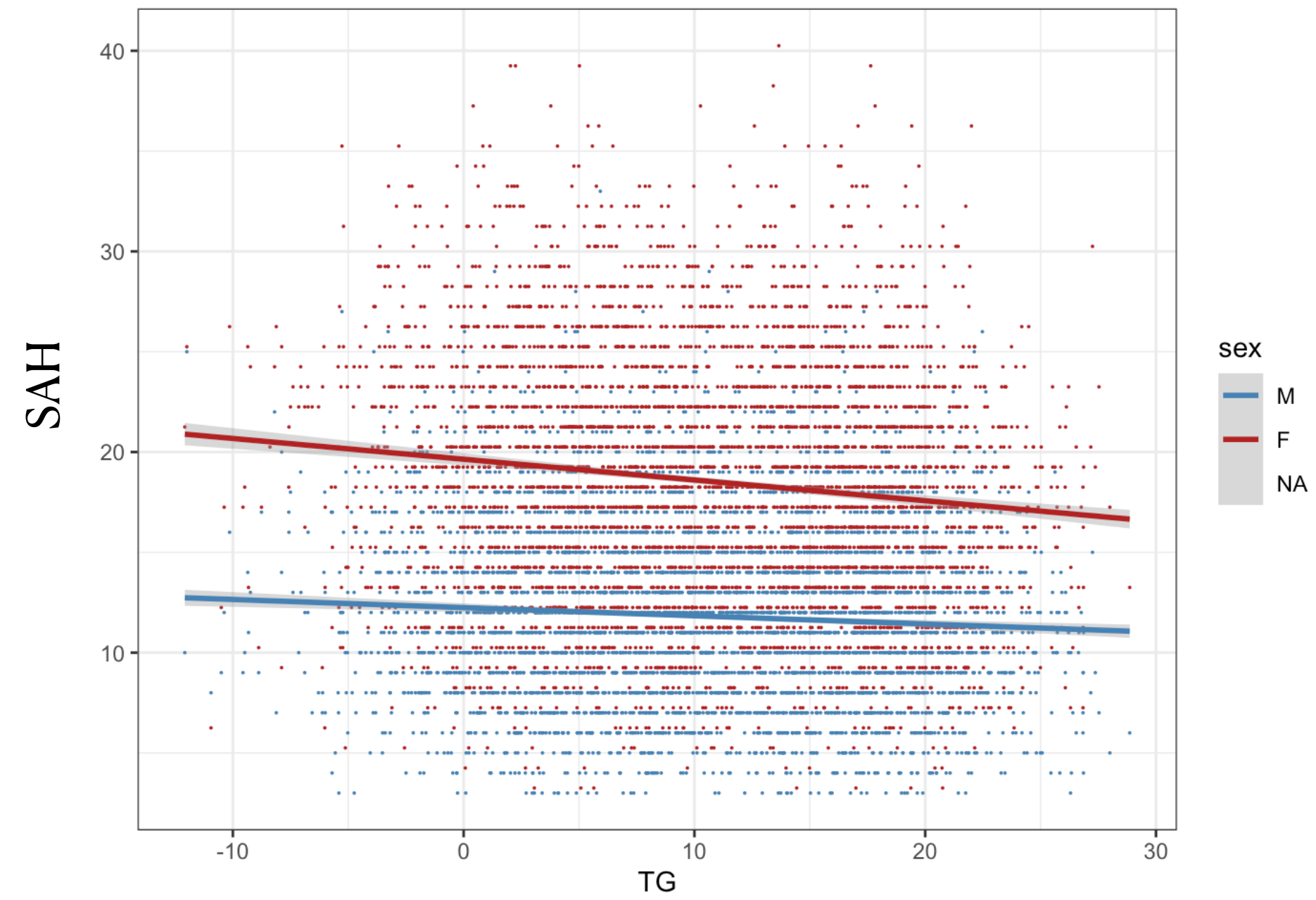

**B** Humidity and aSAH incidence

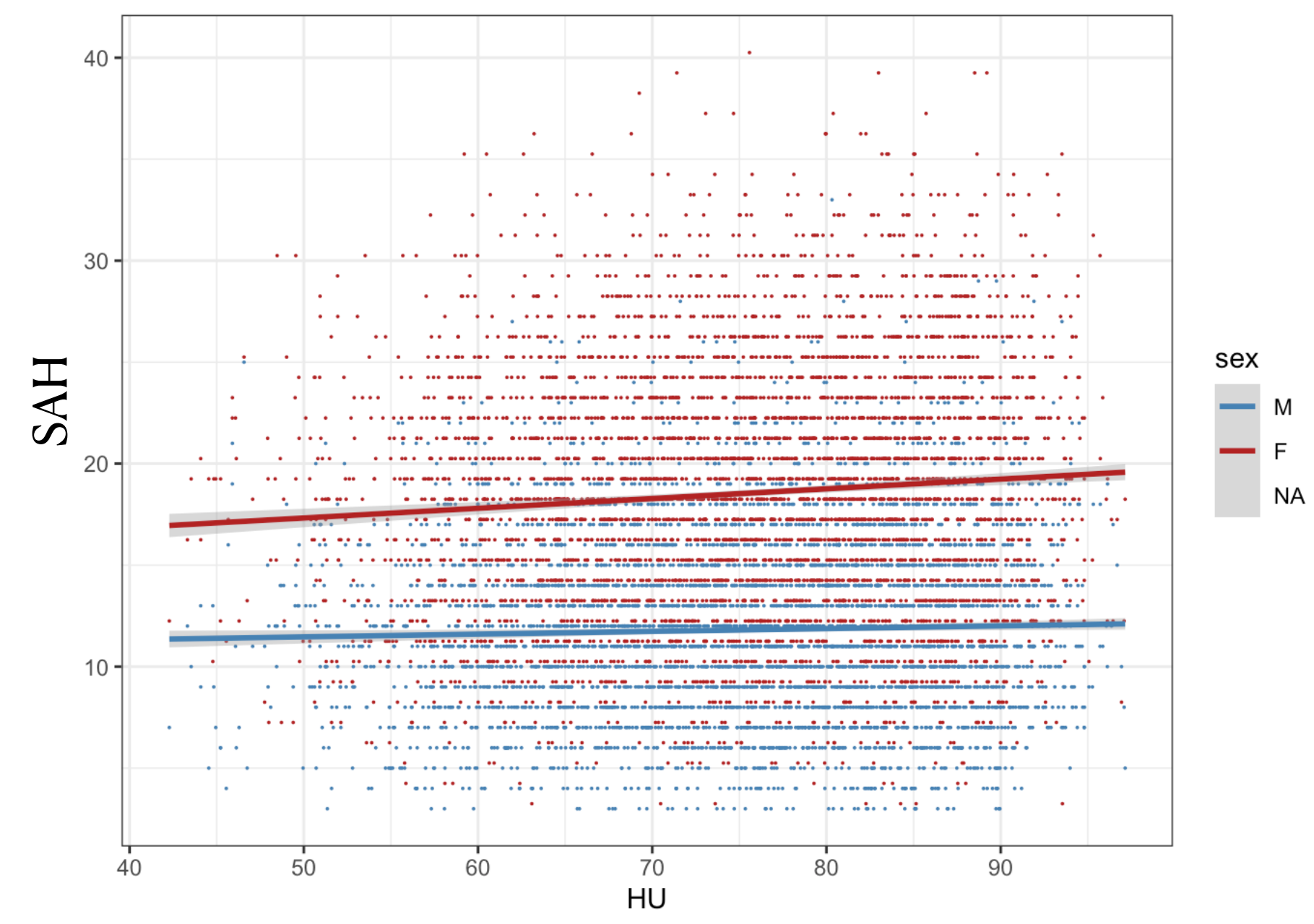

**C** Air pressure and aSAH incidence

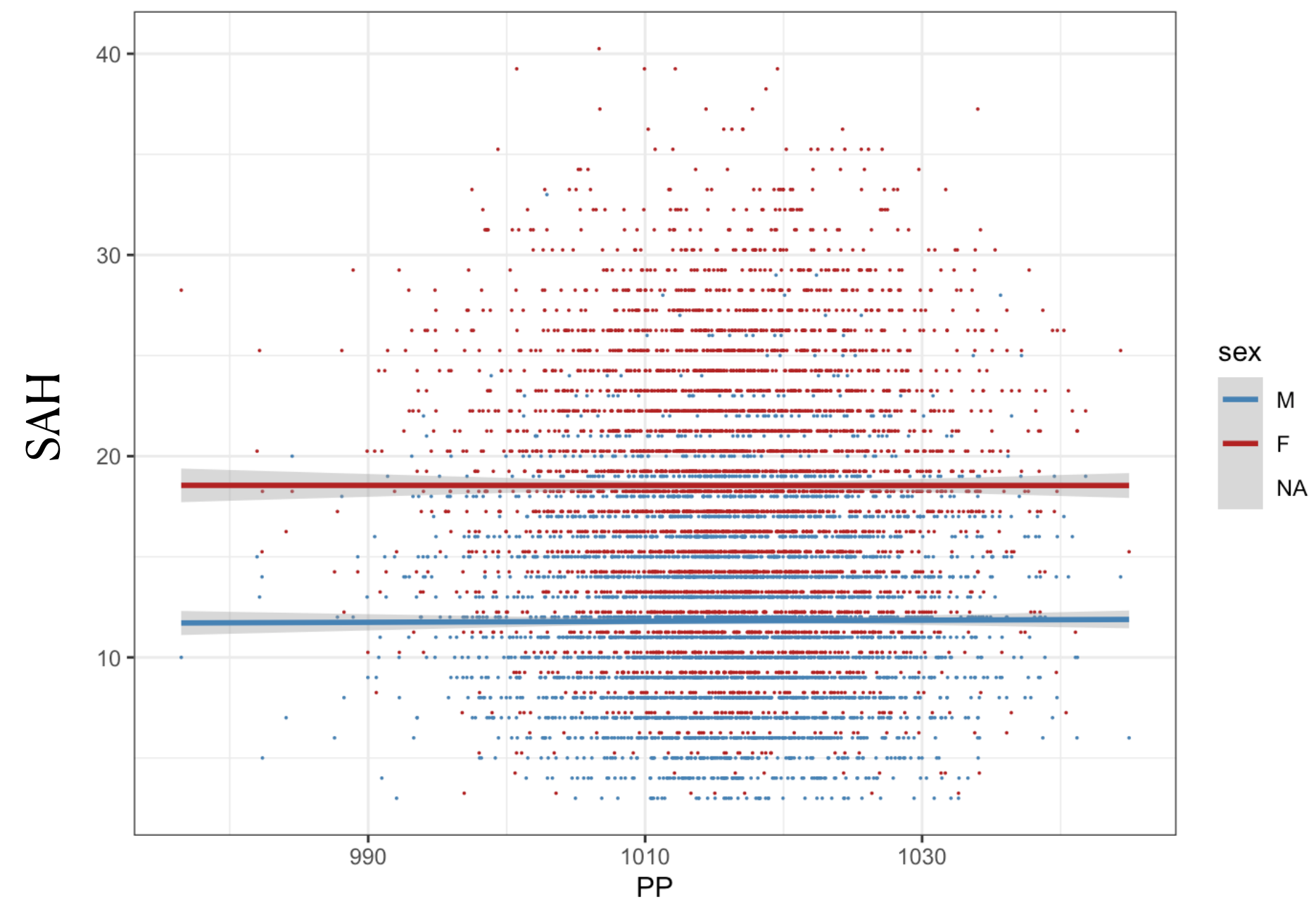

Supplement: Supplementary file 1 — Additional file 1: Supplemental Figure S1. Environmental factors associated with seasons of the year and SAH rate. A A rise of external (environmental) temperature is associated with a decrease of SAH rate. B Higher humidity leads to decrease of SAH rate. C Air pressure differences are not influencing the rate of SAH. [file 41016_2023_322_MOESM1_ESM.pdf]
